# Ulnar Nerve Injury Following Open Carpal Tunnel Release: A Case Report

**DOI:** 10.7759/cureus.32477

**Published:** 2022-12-13

**Authors:** Ahmad S Ashi, Khalid T Alghamdi, Sara Aljohani, Luma Qutub, Meshal F Alghamdi, Layan Kutub, Hussam Kutub

**Affiliations:** 1 Microbiology, King Saud Bin Abdulaziz University for Health Sciences College of Medicine, Jeddah, SAU; 2 College of Medicine, King Saud Bin Abdulaziz University for Health Sciences, King Abdullah International Medical Research Centre, Jeddah, SAU; 3 Infectious Diseases, King Saud Bin Abdulaziz University for Health Sciences College of Medicine, Jeddah, SAU; 4 College of Medicine, Batargi Medical College, Jeddah, SAU; 5 College of Medicine, King Saud bin Abdulaziz University for Health Sciences, King Abdullah International Medical Research Centre, King Abdulaziz Medical City, Ministry of National Guard Health Affairs, Jeddah, SAU; 6 Neurosurgery, Fakeeh College for Medical Science, Jeddah, SAU; 7 Neurosurgery, King Abdulaziz Medical City, Ministry of National Guard Health Affairs, Jeddah, SAU

**Keywords:** ulnar nerve injury, open carpal tunnel release, ulnar nerve palsy, ulnar nerve, carpal tunnel syndrome

## Abstract

Carpal tunnel syndrome (CTS) is one of the most common peripheral nerve diseases. It is managed medically and if not, resolved by surgical procedure. Decompression of the carpal tunnel is considered the definitive treatment. There are multiple complications after this procedure, which can be classified into three categories: (I) persistent, (II) recurrent, or (III) new symptoms, and ulnar nerve palsy after decompression of the carpal tunnel is a rare complication. In this study, we present a case of carpal tunnel decompression, which was complicated by ulnar nerve palsy, which exacerbated a pre-existing chronic ulnar nerve injury. We also explore the possible causes that may have led to this outcome.

## Introduction

Carpal tunnel syndrome (CTS) is the most common peripheral nerve condition in the upper limb, with a projected 10% risk of developing it throughout life. Patients can suffer from pain and tingling sensation along the distribution of the median nerve and thenar muscle atrophy [1]. A large epidemiological study in the United States found that the prevalence of CTS among workers was 7.8%, with higher rates reported in females and older individuals [2].

There are several treatment options for CTS, but the most frequently used is open carpal tunnel decompression, which results in substantial relief of the symptoms of CTS [3]. Complications of open carpel tunnel decompression occur at a rate of 12%. They can be categorized into the persistence of preoperative symptoms, primarily due to improper release of the flexor retinaculum, and new postoperative symptoms, which are commonly iatrogenic injury to median nerve branches [4]. Injury to the ulnar nerve is seldom reported in the literature as a complication of open carpal tunnel decompression. Here, we present a case of ulnar nerve injury following open surgical decompression of the median nerve in a patient presenting with a claw hand.

## Case presentation

**Figure 1:**
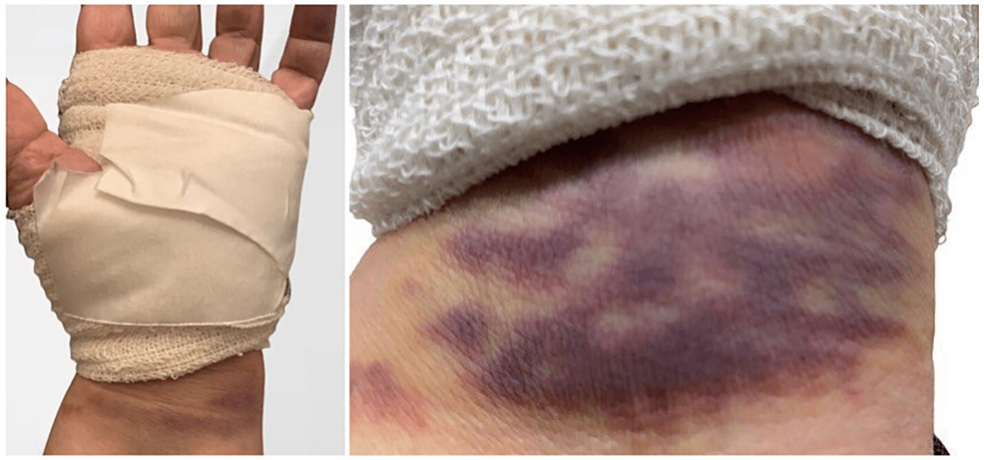
Post-surgery, the patient suffered from a moderate hematoma that appeared at the palmar side of the wrist

**Figure 2:**
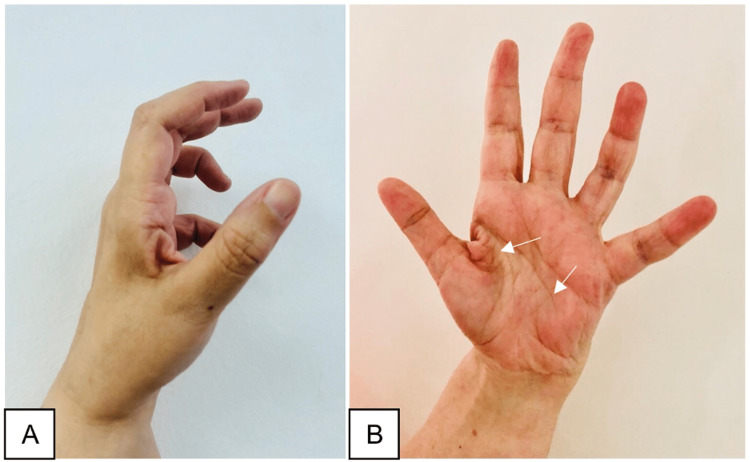
Six months post-operation, the lateral view of the hand (A) showed a claw hand, and the anterior view (B) showed generalized atrophy in the hand muscles sparing the thenar muscles

A 59-year-old female, who is a known case of surgically treated carpal tunnel syndrome in the right hand five years earlier presented with numbness, paresthesia, and pain in the thenar region of her left hand and weakness of grip for one month. She tried medical therapy, such as steroid injections and oral steroids, but showed no notable improvement. The patient underwent a carpal tunnel release procedure under general anesthesia for the left hand. The procedure was complicated with a moderate spike in blood pressure and bleeding during the surgery. The surgery took as twice as long as scheduled. Ten days post-surgery, while changing the dressing, the surgeon found a hematoma in the palmar half closer to the wrist, which could lead to compression over the area. It healed over the next month (Figure 1). Moreover, the patient complained of an inability to abduct fingers. As the dressing was slowly taken off over three weeks, she noticed it evolving into generalized weakness in the hypothenar region, with tingling and pain. Moreover, the hand would naturally fall into a claw-like position when resting (Figure 2).

Investigations

Upon the discovery of these findings, a nerve conduction study was performed and showed electrodiagnostic evidence of distal motor axonal ulnar neuropathy in the left hand. When it came to localization, a drop in flexor dorsal interosseous (FDP) compound muscle action potential (CMAP) with relative sparing of the abductor digiti minimi (ADM) CMAP suggested localization at the wrist. However, chronic changes on electromyography (EMG) alongside clinical involvement of proximal muscles indicate pathology at the level of the elbow as well, which was due to old trauma. The impression of possible double pathology, a chronic pathology at the level of the elbow, and an acute pathology postop at the level of the wrist.

## Discussion

Open carpal tunnel release is a safe and highly effective procedure to relieve the symptoms of CTS. However, as with any procedure, it has complications [3]. The complications can be categorized into three classes, which include: (I) persistent, (II) recurrent, and (III) the development of new symptoms. Persistent complications are considered the most frequent one; it accounts for 7% to 20%. Recurrence of the symptoms after the surgery after a few months is reported to be upto 6%. The third class of complications is the development of new symptoms. It usually happens as an iatrogenic complication, but fortunately, it accounts for only 1% [5]. Direct ulnar nerve injury may occur during an open carpal tunnel release because of the nerve’s anatomical adjacency, but it is considered a rare complication. In our patient, the investigation showed that the patient has a combination of chronic and acute ulnar nerve pathology, which, to our knowledge, has not been reported before.

**Figure 3:**
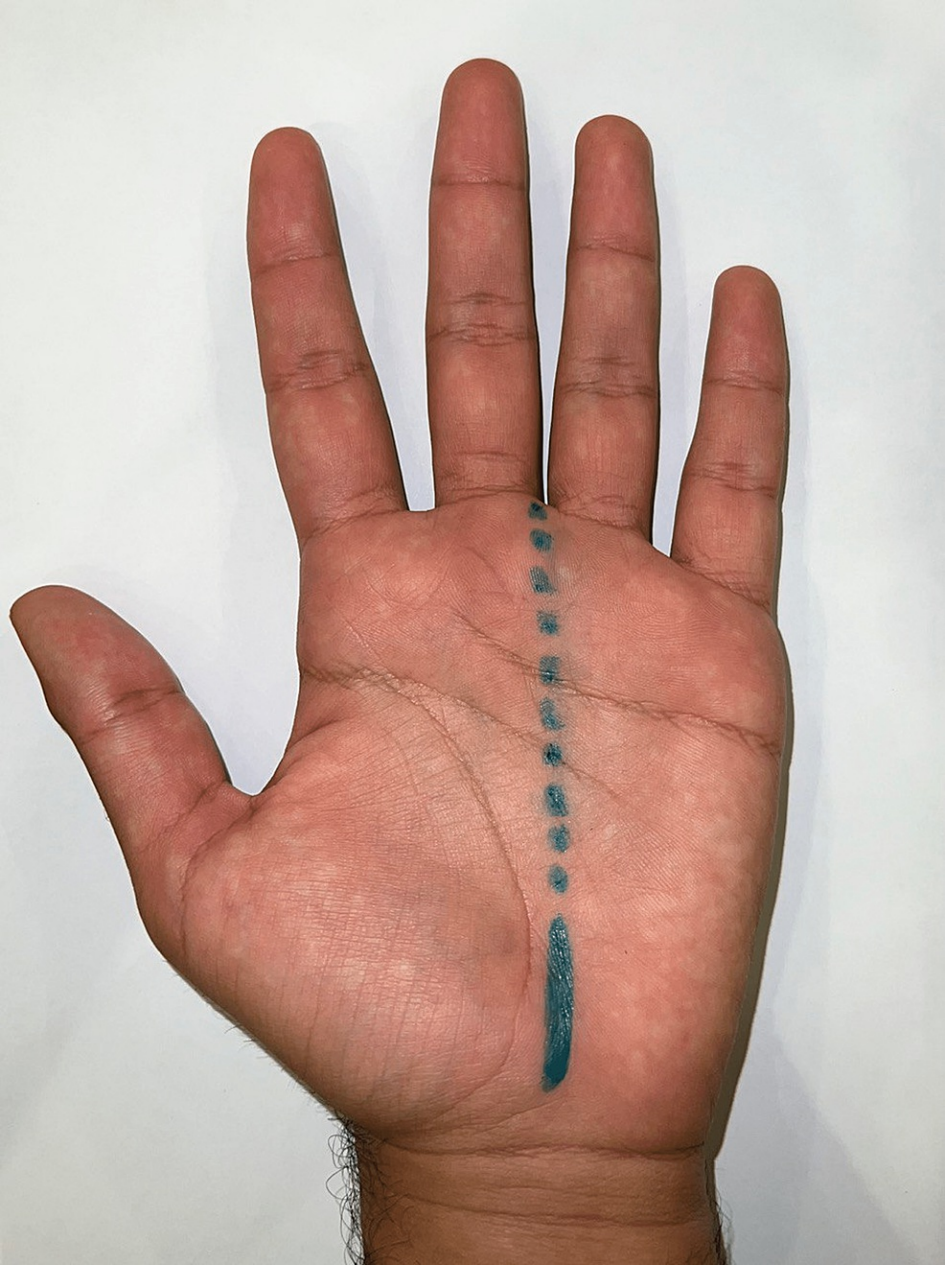
Example of the optimal site and orientation for an open carpal tunnel release incision

A fair understanding of the ulnar nerve’s course is needed for the recognition of possible sites of damage from incisions and where they may manifest. As described by Polatch DP et al., the course begins underneath the flexor carpi ulnaris muscle, where the ulnar nerve passes into the forearm. It gives rise to a palmar cutaneous branch, before giving rise to a dorsal sensory branch This takes place before passing Guyon’s canal. There, a bifurcation takes place into two branches: a deep motor branch, medially, which supplies the hand’s smaller muscles, and a superficial branch, which is sensory [6]. There have been previous reports of deep motor branch injuries, which anatomically described the incisions used in open carpel decompression. This includes an incision in line with the ring finger [7]. Moreover, another report exists where the surgeon opted for an incision located 1 cm medially and parallel to the thenar crease, which distally went on 1 cm medial to the center of the palm (Figure 3) [8].

A case reported in 2008 by P Yoong was more similar to ours, as it followed the classical carpal tunnel incision, which points to the radial side of the ring finger but was complicated by severe bleeding intraoperatively, as compared to our case in which the bleeding was mild and the orientation was overall less radial [9].

Injury of the deep motor branch of the ulnar nerve postoperative is rarely reported, with many different causes across past case reports. It is possible in our case that the intraoperative bleeding may have not been adequately resolved, which lead to the hematoma formation and the subsequent ulnar nerve injury. Moreover, the controversy revolving around the optimal orientation of the carpal tunnel makes it difficult to rule out a dissection of the ulnar nerve distally, unless the forearm is explored surgically [10,11].

## Conclusions

Although ulnar nerve palsy is a rare complication, it should be under consideration following CTS decompression surgery. Therefore, a full neurological examination after the procedure is necessary, not only a limited examination of the median nerve. It is difficult to say with certainty which one factor is the sole cause of an ulnar nerve injury. However, we propose that having a large hematoma post-surgery should be looked at and managed before it can lead to nerve injury. In addition to that, such an injury may be aggravated by having a combination of acute and chronic ulnar nerve neuropathy as a complication, which is rare but can still be seen as demonstrated in our case. Therefore, conducting further in-depth studies on the topic is required to have a better understanding of the pathophysiology and how to avoid such a complication.
